# Modeling and Optimization of the Adsorption of Cr (VI) in a Chitosan-Resole Aerogel Using Response Surface Methodology

**DOI:** 10.3390/gels9030197

**Published:** 2023-03-04

**Authors:** Jean Flores-Gómez, Victor Hugo Romero-Arellano, Milton Vazquez-Lepe, Álvaro de Jesús Martínez-Gómez, Juan Morales-Rivera

**Affiliations:** 1 Departamento de Agua y Energía, CUTonalá-Universidad de Guadalajara, Av. Nuevo Periférico No. 555, Tonalá 45425, Jalisco, Mexico; 2 Departamento de Ciencias Básicas y Aplicadas, CUTonalá-Universidad de Guadalajara, Av. Nuevo Periférico No. 555, Tonalá 45425, Jalisco, Mexico; 3 Departamento de Ingeniería de Proyectos, CUCEI-Universidad de Guadalajara, José Guadalupe Zuno 48, Zapopan 45100, Jalisco, Mexico; 4 Departamento de Ingeniería Química, CUCEI-Universidad de Guadalajara, Blvd. M. García Barragán 1421, Guadalajara 44430, Jalisco, Mexico

**Keywords:** aerogel, chitosan/resole, adsorption, Cr (VI), response surface methodology, computer modeling, optimization

## Abstract

In this paper, a model for Cr (VI) removal and optimization was made using a novel aerogel material, chitosan-resole CS/R aerogel, where a freeze-drying and final thermal treatment was employed to fabricate the aerogel. This processing ensures a network structure and stability for the CS, despite the non-uniform ice growth promoted by this process. Morphological analysis indicated a successful aerogel elaboration process., FTIR spectroscopy corroborated the aerogel precursor’s identity and ascertained chemical bonding after adsorption. Owing to the variability of formulations, the adsorption capacity was modeled and optimized using computational techniques. The response surface methodology (RSM), based on the Box–Behnken design using three levels, was used to calculate the best control parameters for the CS/R aerogel: the concentration at %vol (50–90%), the initial concentration of Cr (VI) (25–100 mg/L), and adsorption time (0.3–4 h). Analysis of variance (ANOVA) and 3D graphs reveal that the CS/R aerogel concentration and adsorption time are the main parameters that influence the initial concentration of CS/R aerogel metal-ion uptake. The developed model successfully describes the process with a correlation coefficient of R^2^ = 0.96 for the RSM. The model obtained was optimized to find the best material design proposal for Cr (VI) removal. Numerical optimization was used and showed superior Cr (VI) removal (94.4%) under conditions of a CS/R aerogel concentration of 87/13 %vol, with an initial concentration of Cr (VI) of 31 mg/L, and an adsorption time of 3.02 h. These results suggest that the proposed computational model can obtain an effective and viable model for CS material processing and for optimization of the uptake of this metal.

## 1. Introduction

Finding effective methods to remove heavy metals has become essential and research with functionalized materials in this area is progressing [1]. Various material composites have been used as methods to manage heavy metals in wastewater; however, adsorption [2] has established itself as a systematic method that is particularly interesting because of its efficiency, selectivity [3], and easy operation. Historically, the high cost of the adsorption material has limited its use, but recently bio-adsorbents that are suitable for this technique have been proposed, [4]. Aerogels are promising advanced materials, well known for their ultra-light and highly porous properties [5,6], that are commonly made from gel through multiple drying methods [7]. When the aerogel is a crosslinked material from a gel precursor, the solvent extraction ensures a large specific surface area, as this feature is essential for adsorption potential [8]. However, the definition of aerogel is controversial: they are solids, with meso- and macro-pores, with nanometer scale porosity but 95% of the phase is gas [5]. Complex processing and drying methods have limited the use of aerogel [9]. In recent years, with the increasing concern for the environment, eco-friendly or natural materials have been used as precursors of many functional materials, such as aerogels [10].

Since the beginning, aerogels have been manufactured with a large range of materials, such as inorganic materials, with carbon [11,12,13], and polymer-based materials [14]; now, a sustainable and cost-effective approach is to use chitosan, a natural polysaccharide that is attractive for aerogel formulation [5,9,15]. The properties of chitosan (CS) are well known and include biodegradability, crystallinity, biocompatibility, and abundance of the polymer. Furthermore, on the surface, the hydroxyl and amine groups are free to uptake anions, which is a highlight for heavy metal applications [16,17]. The general pathway for chitosan aerogel formation includes a sol–gel reaction, where the aqueous solution of chitosan transitions to gel; this determines the formation of a three-dimensional (3D) porous material which is occasionally used as a template for composite materials [18,19]. It is critical that factors such as pH, and temperature are controlled. Then the aging treatment is applied, where the gels are immersed under controlled conditions to enhance mechanical strength, and to ensure the internal network becomes structured. The ranges of times and solvents used are large [20,21]. The formation mechanism of the aging process remain unclear and limited to speculation. Further research needs to be conducted on the physical and chemical changes in the gel [6]. Another crucial factor is that the type of final aerogel structure also depends on the mold where the gelation process takes place, that being an essential determinant of ice crystal growth direction [20]. The rate of freezing and the drying method to achieve the removal of the internal solvent, i.e., supercritical drying or freeze-drying, also impact the outcome of the aerogel, which further complicates the processing. The response surface methodology (RSM) is a combination of mathematical and statistical methods for improving the process variables or evaluating the significance of the parameters of complex interactions. It has the advantage of analyzing multiple factors with minimal take-up of experiment runs, which is important because, typically, optimizing a process requires several experiments, multiple runs, long intervals of time, and costs a lot. The design of experiments with a statistical base can drastically reduce the number of experiment runs and provides a model that can describe the process with a polynomial equation and optimize the process involved [22,23,24]. The RSM is an effective tool for determining and understanding the desired effects. These results can be optimized using the model obtained with a polynomial equation describing the response surface.

Seyed et al. [2] proposed a hollow chitosan fiber and used the RSM to analyze the influences of the main operating parameters; Santos-López et al. [25] also obtained CS aerogels that maintained the chemical identity of the CS after processing, and Guo et al. [26] accomplished polydopamine-modified chitosan aerogel beads with a sorption performance of 374.4 mg/g of Cr (VI).

In the processing of CS aerogels, this study encompassed CS-R aerogels with resole (R): these liquid resins are obtained from a condensation polymerization synthesis based on phenol with an excess of formaldehyde [14,15]. A facile strategy for sorbent preparation to obtain functional CS/R aerogel with a xylem-like structure involves dispersal of these resins on a CS solution to promote a sol–gel reaction, followed by a freeze-drying process and a then a thermal treatment for the curing of the dispersed thermosetting polymer, as [15]. Enlarging the size of the used adsorbent, as a strategy to propose an industrial application, is essential for this study. Owing to the variability of the formulations of different volume combinations, an RSM design analysis was used to verify the model, and further optimize Cr (VI) adsorption, based on CS/R aerogel percentage concentration, initial concentration of Cr (VI), and adsorption time, using a three-level three-factor full-factorial Box–Behnken design (BBD). The removal of Cr (VI) was proposed due to its known carcinogenicity to humans, it is considered one of the significant chemical contaminants by the World Health Organization (WHO) [26], and due to the ease of its measurement by spectroscopic techniques.

## 2. Results and Discussion

### 2.1. RSM Modeling and Optimization

In this research, a Box–Behnken design (BBD) with three levels was used for modeling and approximating the adsorption of Cr (VI) with the CS/R aerogel. An experimental matrix with three factors was developed: (A): chitosan/resin concentration at vol (50/50, 75/25, and 90/10 %) (B): initial concentration of Cr (VI) (25, 50, and 100 mg/L); and (C): adsorption time (0.5, 1.5 and 4 h). The response variable was the adsorption of Cr (VI) (Y_1_). A second-order polynomial Equation (1) was established using the three independent variables to predict the response values. *Y* denotes response (adsorption of Cr (VI), %); b_0_ is the intercept value; *b*_i_ (_i_ = 1,2,3,…,n) signifies the first model quantities for X_i_; b_ii_ signifies the quadratic coefficients of X_i_; b_ij_ are the correlation factors for *X*_i_ *X*_j_; and ɛ is the random error. Design-Expert software 10.0.1 (Stat-Ease Inc., Minneapolis, MN, USA) was used for all numerical assessments, analysis of variance (ANOVA), and numerical optimization of the RSM model [27]. The independent variables and their three levels appear in Table 1.

(1) $$Y=b_{0}+\overset{k}{\underset{i=1}{\sum }}b_{i}X_{i}+\overset{k}{\underset{i=1}{\sum }}b_{ii}X_{i}^{2}+\overset{k}{\underset{i=1}{\sum }}\overset{k}{\underset{j=1}{\sum }}b_{ij}X_{i}X_{j}+\varepsilon$$

### 2.2. Characterization of CS/R Aerogel

Characterization of material properties such as morphology, density, and chemical composition were performed on the CS/R aerogel in order to understand the manufacturing process.

#### 2.2.1. Morphology of CS/R Aerogel

**Figure 1:**
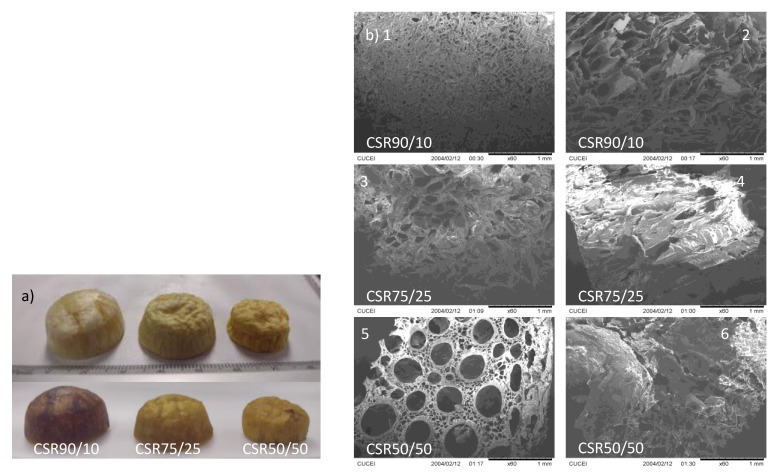
Chitosan/resin concentration, %vol. (**a**) CS/R aerogel before adsorption (above) and after adsorption (below); (**b**) SEM micrographs, 60X, of CS/R aerogel (1, 2) 90/10, (3, 4) 75/25, and (5, 6) 50/50.

The morphological transition from the sol–gel CS/R dispersions to CS/R aerogels was observed; these could be molded into arbitrary shapes depending on the size required; in this case, they were molded into bullet shapes using silicon molds, to avoid further reaction with R. Figure 1a, shows a photograph of the aerogels after processing and the thermal treatment; these were rigid and brittle and all CS/R aerogels were equipped with a three-dimensional non-uniform network structure, The structure of the adsorbent was maintained successfully throughout the solvent extraction using the freeze-drying technology. The R solution was diluted in ethanol; this, in conjunction with the diluted CS, promotes a solvation effect favoring the interactions of the polysaccharides chains and leading to a gel [25]. This, and the further molecular conformation and network formation of CS indicates that the combination of precursors solutions was uniformly dispersed.

This 3D structure was beneficial for adsorption, allowing a successful diffusion of the metal; this was verified by the FTIR analysis. In Figure 1a, CSR75/25 and CSR50/50 show the decreasing size and diminished structure of the material in comparison with its dry weight. The average of CSR50/50 aerogel was 0.3682 g, which is almost 42% more than the 0.1534 g of CSR90/10 aerogel which is more extensive and has lighter density due to the decreased R in the formulation, which is probably associated with the structure of the chitosan template. In addition, it is evident in the lower image in Figure 1a that, after adsorption, the physical structure was maintained. The network structure was not destroyed even by swelling and stirring, which demonstrates the stability and tolerance of the aerogel; a dark brown coloration was seen, corresponding to metal attached to the surface of the aerogel. This coloration result after Cr (VI) adsorption was also obtained by Wang et al. [28] on β-cyclodextrin/chitosan/hexamethylenetetramine (β-CD-CS@HMTA) aerogel beads. From the SEM micrographs, the internal structure network could also be seen; the methodology process produces a non-uniform growth from the ice crystal due to the concentration of R in the aerogel to ensure overall mechanical strength. The final, thermal treatment, step accomplishes a type of aging of the structure without the solvent. This methodology produces an aerogel that is easy to process and can maintain its properties and characteristic features [13,29]. In Figure 1b, a superior cut of the CS90/R10 aerogel allows us to see the density and uniform porosity; these were probably chitosan molecules, which have smaller dimensions and generate more compact structures [25,30], Figure 1b with a cross-section cut, reveals the formations of channel like and xylem-like irregular tubular growths; this feature was present in the majority of samples generated using the CS template and is a characteristic formation with chains of polysaccharides [21]. A similar morphological structure was observed in Figure 1b, where the pore density and uniformity decrease but the tubular growths remain visible. In contrast, the CSR50/50 aerogel seen in Figure 1b, with a considerable increase in R, does not show uniform pore structure, and bubbles have formed. Although porosity was maintained, the tubular structure is no longer so well-defined. Volume and density were evaluated with a gas pycnometer and the results are shown in Table 2.

The results in Table 2 are consistent with the morphology observed in Figure 1a, indicating that the narrow pore density space decreases with increasing resole concentration; density values were also similar to other reports, such the one obtained for Meng et al. [31], of 0.0662 g/cm^3^ with chitosan/cellulose aerogel.

#### 2.2.2. FTIR of CS/R Aerogel

**Figure 2:**
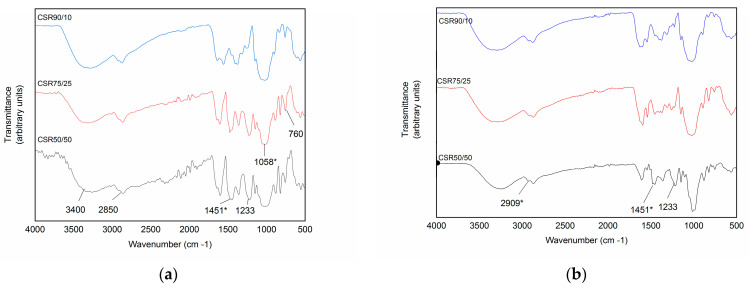
FTIR Spectrum comparison: (**a**) CS/R aerogel after processing; (**b**) CS/R aerogel after adsorption of Cr (VI). * Indicates significant changes in the FTIR spectrum before and after adsorption.

FT-IR analysis was carried out to confirm the functional groups on the surface of the CS/R aerogel; the FTIR spectroscopy of CS/R aerogels at 90/10, 75/25, and 50/50 percentage concentrations are shown in Figure 2a; characteristic peaks of the precursor material were found.

Prominent characteristic bands of chitosan were seen in the case of highly deacetylated CS; it was noted that a band corresponding an –O-H overlaps with an –NH observed at 2850 cm^−1^, indicating a high density of amine units. Peaks corresponding to amide II at 1550 cm^−1^ and CH_2_ at 1451 cm^−1^ were also present and there was a band corresponding to saccharide at 760 cm^−1^ in the fingerprint zone [15,25,32,33]. In the case of R, a polymeric (O-H) was found at 3400 cm^−1^, a benzene ring (C=C) at 1600 cm^−1^, as well as an ether bridge tension (C-O-C) at 1058 cm^−1^; these demonstrated that R had been successfully introduced into CS aerogels. The R polymer overlaps with the N-H bends for 3300 s cm^−1^ and intensifies the peaks in the 1800–400 cm^−1^ range due to the methylene ether bridges expected as a result of curing. After adsorption, in Figure 2b, the peak observed at 2850 cm^−1^ shifts to 2909 cm^−1^; in addition, the peaks at 1451 cm^−1^ and 1233 cm^−1^ are weakened, in comparison to the spectrum produced without metal uptake, indicating that NH- and NH_2_- contributed to the adsorption of Cr (VI) and decreased number of methylene ether bridges [26,28].

### 2.3. RSM Modeling

An empirical model was proposed to understand the interactive correlation between the response and process variables; the model obtained in terms of actual factors is given below in Equation (2).

(2) $$Y_{1}\left(\%\right)=57.02+30.75A-4.93B-8.67C-8.35AB-3.17AC-28.56BC-0.72A^{2}-5.59B^{2}-2.63C^{2}\;$$

ANOVA analysis, shown in Table 3, was employed to determine the fitting of the developed model in conditions of coded factors. F and *p* values, the degrees of freedom, and a value for p below 0.05 indicate the importance of that model term [34]. In this study, two of the main parameters were significant for the RSM model, according to *p*-value evaluation, revealing (*A*) chitosan/resole percentage concentration as the most transcendental parameter for this process and (*C*) adsorption time as an essential factor for the RSM model. The term (*BC*) has a *p*-value < 0.05 and was very significant for this model. All other results has *p* values > 0.05, indicating that these are not significant for the RSM model; at a confidence level of 96.9%, the, R^2^ (0.9659) and R^2^ adjusted (0.93) results suggest excellent performance of the RSM models [35].

Table 4 displays 17 Cr (VI) removal experiments using chitosan/resole aerogel material under different parameters, applied to a three-level three-factor Box–Behnken design (BBD) RSM. In this study, the results were consistent with other works reported in the literature [23], and the RSM was feasible for CS material optimization processing [36].

#### 2.3.1. Effects of the Variables on Cr (VI) Adsorption

**Figure 3:**
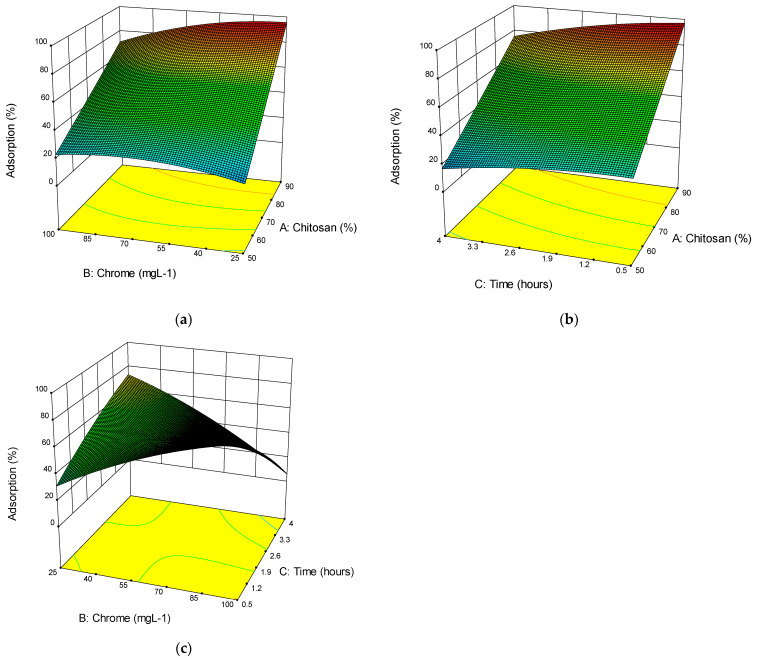
Three-dimensional figures for Cr (VI) adsorption (**a**) chitosan vs. chrome at 2.3 h adsorption time, (**b**) time vs. chitosan at 62.5 mg/L. Initial concentration of Cr(VI) (mg/L), and (**c**) chrome vs. time at 69/31% vol chitosan/resole concentration volume (%).

Based on data from Table 3, tridimensional graphics, shown in Figure 3a–c, were produced to evaluate the interactive effects of all variables: (a) chitosan/resole concentration (% vol), (b) chrome (mg/L), and (c) time (h). The results of these graphs and the conclusions of the main variables are analyzed in the following paragraphs.

#### 2.3.2. Effect of Chitosan/Resin Concentration

To analyze the effect, the adsorption, or % uptake, was used as a process response. The CS and R percentage concentration influenced the amine groups grafted onto the aerogel surface, affecting the sorption capacity of the aerogel, with increasing CS concentration, more sorption groups could be on the surface. More R led to an increase in the mechanical strength of the aerogel, resulting in the removal of possible binding sites for anions, which decreased the sorption capacity. As shown in Figure 3a, the sorption capacity of CS/R aerogel increased with an increasing percentage of CS. Concentration of 80% and above were needed to remove low Cr concentrations; increasing the Cr concentration decreases the adsorption, the maximum uptake being a 94.4% removal. Mao et al. [23] also concluded that the RSM is a good predictor of the influence of dosage on material processing.

#### 2.3.3. Effect of Initial Concentration Cr (VI)

To study the effect of the initial concentration, another graph or surface response was made, shown in Figure 3b. It evident that this factor was affected by CS/R percentage concentration and had a positive effect on adsorption at CS volumes greater than 80%; this was expected, because a larger surface area CS ensures more significant adsorption of metal ions. In addition, a high initial concentration is more likely to overcome the mass transfer resistance because of the increased effective area of contact with the aerogel [28]. This consistent with other reported results that show that modified CS improved adsorption compared to unmodified CS [37].

#### 2.3.4. Effect of Adsorption Time

The effect of adsorption time is shown in Figure 3c. A rapid removal of Cr (VI) occurred within 0.3 to 2 h, then slow adsorption subsisted until 4 h. In comparison, equilibrium increased with the increase in initial concentration. The reason was that the adsorption sites on the aerogel surface were rapidly saturated in the experiments with high Cr (VI) concentration, which meant that. ions needed to be further transferred into the internal channels of the aerogel and adsorbed onto these free sites; this takes time, so the process is slower. In order to evaluate the maximum sorption potential of the CS/R aerogel formulations kinetics studies of factor A, in combination with factor B, were conducted under the same conditions as the original experimental run, for 24 h. Then, subsequent models pseudo-first-order and pseudo-second-order were used to evaluate the kinetics. Equations (3) and (4) are described as follows:

(3) $$ln(q_{e}-q_{t})=lnq_{e}-k_{1}t\;\;\;$$

(4) $$\frac{t}{q_{t}}=\frac{1}{k_{2}q_{e}2\;\;}+\frac{t}{q_{e}}$$

where *q_e_* and *q_t_* (mg/g) signify the quantity of Cr (VI) on the CS/R aerogel at equilibrium and time *t* and *k*_1_ (min^−1^) and *k*_2_ (g(mg min)^−1^) corresponds to rate constants of pseudo-first-order and pseudo-second-order, respectively.

**Figure 4:**
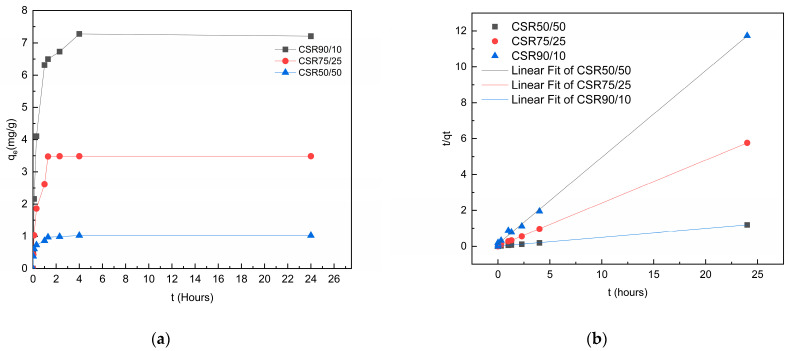
Kinetic study of CS/R aerogels in combination with initial concentrations (**a**) Influence of adsorption time on Cr (VI) removal with 25 mg/L as initial concentration; (**b**) pseudo-second-order kinetic model of Cr (VI) adsorption with 100 mg/L as initial concentration.

The kinetic study is essential for practical applications such as operation control [23]; The adsorption kinetic model curve is shown in Figure 4a. Equilibrium was attained in the first 4 h, leading to a concave curve with a strict plateau being visible. The corresponding kinetics of Cr (VI) are also listed in Table 5 and Figure 4b; The curves fitted a pseudo-second-order model with a higher correlation coefficient (R^2^) than those with the pseudo-first-order model, which shows that chemisorption was limiting the adsorption processes.

This contrasts with Figure 3c, in which it can be seen that the other two factors did not influence the contact time, because the equilibrium time was almost the same in both experiments; this is consistent with previous work [38] that estimated 300–400 min to reach equilibrium using modified CS.

### 2.4. Optimization Process

According to Table 6, the results of RSM numeric optimization reveal the ideal CS/R aerogel parameters for maximizing Cr (VI) adsorption.

These results show progress in CS/R aerogel adsorption compared with the 17 experiments shown earlier in Table 4. The test were performed in triplicate for reproducibility in the laboratory.

## 3. Conclusions

In this work, a CS/R aerogel was fabricated using a sol–gel reaction of a CS solution with a resole solution via phenol-formaldehyde polymerization; this accomplished a simultaneous self-polymerization of the CS gel dispersed with liquid resole. The aerogel shows a highly porous structure and large surface area, facilitating the removal of Cr (VI) ions. The morphological analysis also showed that the proposed processing includes a thermal treatment as the final step in order to broaden its application by enhancing acid resistance and giving mechanical strength to the internal network. FTIR analysis shows that the main chemical species, chitosan and resole, were present in the different formulations of the CS/R aerogel; it also established a chemical bond with NH and NH^2^ species after adsorption. Furthermore, the method is easy and cheap, representing a simple and reproducible method for manufacturing aerogels of any size. The experimental design established an empirical relationship between the adsorption and independent variables, which was further expressed by the polynomial equation and RSM graphs. The variables of CS/R percentage concentration and adsorption time were related, with the best experimental adsorption capacity being a 95% Cr (VI) uptake. The adsorption kinetics indicated a pseudo-second-order model, suggesting a chemisorption mechanism. The model proposed was effective for material optimization and processing, and the optimal parameters of operation were 87/13 of CS/R aerogel percentage concentration; this is of potential interest as a lower-cost adsorptive material that could efficiently remove other heavy metals. Further research should be carried out, such as measuring the effectiveness of the CS/R aerogel with continuous effluent, or testing its application with other heavy metals, so that we can determine the type of chemical adsorption that occurs and produce a more detailed surface analysis.

## 4. Materials and Methods

### 4.1. Chemicals and Reagents

Chitosan powder (<85%) of deacetylation grade was obtained in a dried powder form (America Alimentos S.A. de C.V, Guadalajara, Mexico). Analytical grade acetic acid (99%), phenol (99%), formaldehyde (37.0%), sodium hydroxide (98%), acetone (99.0%) potassium dichromate (99%), nitric acid (66.0%), 1,5-diphenyl carbazide, HCl (37.0%), and sulfuryl acid (97.3%) were purchased from Fermont and Golden Bell. Deionized water and ethanol were also used.

### 4.2. Fabrication of the Aerogel Process

To fabricate CS-R aerogel, it is necessary to mix the two precursor solutions in a sol–gel reaction. The first solution is the diluted chitosan, at 2% *w*/*v* with acetic acid. This was made with a mixture of weighed chitosan powder with deionized water and acetic acid and allowed to sit at room temperature for three days. The second solution is the diluted resole product of the polycondensation of phenol-formaldehyde (1:3.5) in NaOH at 0.1 M. This method was adapted from [21]. This solution was made by mixing phenol and formaldehyde in NaOH solution for 1 h at 70 °C with continuous stirring, then the pH was adjusted to neutral 7, and the water generated during the polymerization reaction was extracted with a rotary evaporator at 50 °C and 50 mbar for 1 h. Subsequently, the mix was removed, ethanol was added, and then the rotary evaporator was again used for 15 min; subsequently, the liquid resole was removed and refrigerated at −3 to −4 °C to stop polymerization.

#### Formulation and Molding of the CS/R Aerogel

In a syringe, the required amount, based on volume percentage, of the two solutions was mixed, ejected onto the necessary mold, freeze stored in an ultra-freezer at −70 °C and lyophilized (Freeze-Dryer system Ilshin Biobase TFD8501, Komachine, Gyeonggi-do, Korea) for 24 h. Subsequently, a process of curing (> 60°C) or thermal treatment is required to finalize chemical polymerization.

### 4.3. Characterization of the CS/R Aerogel

The characterization of the adsorbent was carried out before and after adsorption. Observing its surface morphology was investigated using a scanning electron microscope (SEM; Hitachi TM-1000, Tokyo, Japan) with a gold coating for 30 s. The volume and density of the pieces were determined by a gas pycnometer (Quantachrome, Ultrapyc 1200e Anton Paar, Graz, Austria). For the verification of chemical identity on the aerogel, a Fourier Transform Infrared Radiation (FTIR) spectroscopy was performed; using attenuated total reflection mode (ATR). These measures were taken at room temperature using an IR spectrophotometer (Alpha II Bruker, Billerica, MA, USA), analyzed in the range of 400–4000 cm^−1^.

### 4.4. Adsorption Studies

Batch Cr (VI) sorption experiments were conducted to analyze the adsorption performance of the adsorbent. The conditions were at 25 −/+ 2 °C, one piece of the aerogel was brought into contact with 50 mL of the required Cr (VI) solution in a 50 mL falcon tube without pH adjustment and collocated in shaking (orbital shaker at 400 rpm). After a definite time and equilibrium had been attained, the adsorbent was separated from the solution. The remaining concentration of Cr (VI) was determined after dilution, measured using diphenyl carbazide, a technique proposed for standard methods [39,40], and analyzed at 540 nm [28] using a UV/VIS spectrophotometer (Jasco v770 spectrometer Tokyo, Japan). The experiments were carried out in triplicate, and average values are reported.

The adsorption capacity of CS/R aerogel, *qt,* was calculated by the following Equation (5):

(5) $$q_{t}=\frac{(C_{0}-C_{t})\;V}{m}$$

where *C*_0_ (mg/L) indicates the initial concentration of Cr (VI) and *Ct* (mg/L) corresponds to its concentration at time *t. V* is the solution volume, and *m* is the adsorbent mass (g).

To evaluate the quality of the adsorption capacity with the RSM, a batch experiment was carried out to determine the level of metal ions in the water at the end of the adsorption treatment. This estimation was made using Equation (6) [36]:

(6) $$R=\frac{Y_{0}-Y}{Y_{0}}100$$

where *R* represents the adsorption efficiency and, *Y*_0_ is the initial value and *Y* is the final adsorption measurement, respectively.

## Figures and Tables

**Table 1:** Independent variables and their levels.

Factor	Parameter	Coded Variables		
		−1	0	1
A	CS/R concentration (%vol)	50/50	75/25	90/10
B	Initial concentration Cr(VI) (mg/L)	25	50	100
C	Adsorption time (h)	0.5	1.5	4

**Table 2:** Volume and density of CS/R aerogels.

Name	Values		
	Weight (g)	ve(cm^3^)	Density d/cm^3^
CSR90/10	0.1400	0.1267	0.0348
CSR75/25	0.1892	0.1459	0.0471
CSR50/50	0.3066	0.1742	0.0763

vt used 4.02 cm^3^, ve = volume of empty space.

**Table 3:** Analysis of variance (ANOVA).

Source	Sum of Squares	df	Mean Square	F Value	*p*-Value
Model	13,405.87	9	1489.54	25.10	0.0002
A: Chitosan/resole concentration at %vol	6623.49	1	6623.49	111.63	<0.0001
B: Initial concentration Cr (VI)	174.85	1	174.85	2.95	0.1297
C: Adsorption time	557.06	1	557.06	9.39	0.0182
AB	301.02	1	301.02	5.07	0.0630
AC	44.81	1	44.81	0.76	0.4136
BC	3729.75	1	3729.75	62.86	<0.0001
A^2^	1.83	1	1.83	0.031	0.8657
B^2^	97.80	1	97.80	1.65	0.2401
C^2^	17.73	1	17.73	0.30	0.6016
Residual error	415.33	7	59.33		
Lack of fit	15.33	3	138.44		
Pure error	0.000	4	0.000		
Cor total	13,821.20	16			

**Table 4:** Box–Behnken design.

Run	Independent Variables				
	X1	X2	X3	Experimental	Predicted RSM
	Chitosan/Resole Concentration (%vol)	Initial Concentration Cr (VI) (mg/L)	Adsorption Time (h)	Cr VI Removal (%)	Cr VI Removal (%)
1	0	0	0	65.87	57.02
2	0	0	0	65.87	57.02
3	1	0	1	91.41	75.75
4	1	0	−1	81.08	93.09
5	1	1	0	90.68	68.18
6	0	0	0	65.87	57.02
7	−1	0	−1	15.97	31.59
8	0	1	1	5.86	6.64
9	−1	1	0	38.78	23.38
10	−1	0	1	30.28	14.25
11	0	0	0	65.87	57.02
12	1	−1	0	85.38	94.74
13	0	−1	−1	56.41	33.84
14	−1	−1	0	0.93	16.54
15	0	−1	1	77.47	73.62
16	0	1	−1	86.00	81.1
17	0	0	0	65.87	57.02

**Table 5:** Kinetic parameters of Cr (VI) adsorption at different initial concentrations and CS/R aerogel ratios.

CS/R Aerogel	C_o_	q_e_, exp	Pseudo-First-Order		Pseudo-Second-Order	
	(mg/L)	(mg/g)	k_1_	R^2^	k_2_	R^2^
90/10	25	7.20	0.0125	0.8926	0.1376	0.9997
	50	8.50	0.0534	0.9011	0.1159	0.9982
	100	20.12	0.0170	0.8339	0.0669	0.9998
75/25	25	3.49	0.0235	0.8334	0.2847	0.9997
	50	6.32	0.0734	0.4513	0.1579	0.9999
	100	4.17	0.0329	0.8782	0.2393	0.9999
50/50	25	1.03	0.0091	0.8378	0.0392	0.9999
	50	1.63	0.0202	0.8288	0.6100	0.9998
	100	2.05	0.0040	0.3896	0.4833	0.9996

**Table 6:** Optimal values for RSM optimization.

Method	Chitosan/Resole Concentration at Vol (%)	Initial Concentration Cr VI (mg/L)	Adsorption Time (h)	Predicted Value	Actual Value
RSM	87/13	31.51	3.02	94.83	94.44

## Data Availability

Data is available by corresponding author upon request.
